# A Model of the Effects of Parental Illness on Youth Adjustment and Family Functioning: The Moderating Effects of Psychological Flexibility on Youth Caregiving and Stress

**DOI:** 10.3390/ijerph18094902

**Published:** 2021-05-04

**Authors:** Giulia Landi, Kenneth Ian Pakenham, Mariagrazia Benassi, Sara Giovagnoli, Eliana Tossani, Silvana Grandi

**Affiliations:** 1 Department of Psychology, University of Bologna, Viale Berti Pichat 5, 40127 Bologna, Italy; mariagrazia.benassi@unibo.it (M.B.); sara.giovagnoli@unibo.it (S.G.); eliana.tossani2@unibo.it (E.T.); silvana.grandi@unibo.it (S.G.); 2 Laboratory of Psychosomatics and Clinimetrics, Department of Psychology, University of Bologna, Viale Europa 115, 47023 Cesena, Italy; 3 School of Psychology, The University of Queensland, Brisbane, QLD 4072, Australia; k.pakenham@psy.uq.edu.au; 4 Italian Society of Environmental Medicine (SIMA), 20149 Milan, Italy

**Keywords:** parental illness, youth caregiving, youth adjustment, family functioning, stress, psychological flexibility

## Abstract

Parental chronic illness may adversely impact youth and family functioning. This study examined a moderated mediation model of the effects of parental illness on youth and family functioning derived from the Family Ecology Framework. Consistent with this model, we predicted that youth caregiving and stress would serially mediate the adverse impacts of parental illness on youth adjustment and family functioning and that psychological flexibility would moderate these mediational mechanisms. A total of 387 youth, with parents affected by chronic illness, completed a questionnaire assessing parental illness severity, youth caregiving and stress, psychological flexibility, youth adjustment (i.e., internalizing and externalizing problems and psychological wellbeing), and family functioning. Path analyses indicated that the adverse effects of parental illness on youth adjustment and family functioning were serially mediated by youth caregiving and stress. Psychological flexibility buffered the adverse effects of these serial mediators on youth internalizing problems and psychological wellbeing. These findings identified three potential intervention targets: youth caregiving, related stress appraisals, and psychological flexibility. Given the large body of evidence showing that acceptance and commitment therapy fosters psychological flexibility, this intervention approach has the potential to address the psychosocial and mental health vulnerabilities of youth in the context of parental illness, which constitutes a serious public health issue.

## 1. Introduction

Parental illness may adversely affect youth and family functioning. It is estimated that approximately 12% to 15% of youth have a parent with a chronic illness [1,2]. Furthermore, given the ongoing increase in numbers of adults living with a serious medical condition worldwide, the number of youth affected by parental illness is likely to steadily rise [3,4]. Parental illness is associated with a significantly higher risk for youth mental and physical health problems, poorer health-related quality of life (HRQoL), and social, educational and employment difficulties that persist well into adulthood [5,6,7,8,9,10,11,12,13,14,15]. Moreover, having a parent with a chronic illness is related to a significantly higher risk for internalizing problems (e.g., depressive, anxiety, and somatic symptoms), externalizing problems (e.g., aggressive and delinquent behaviors), lower life satisfaction, and loneliness [9,10,11,12,13,14,15,16,17]. Compared to peers, the youth of chronically ill parents are also at higher risk of affective dysregulation, stress-related somatic disorders, and weakened immune responses [3,5,16,18]. Parental illness is also related to poorer family functioning, which is associated with more family conflicts, less cohesion, and reduced communication which, in turn, are associated with poorer youth adjustment [3,5,7,19,20]. In order to clarify the inter-relations among factors that exacerbate and ameliorate the adverse effects of parental illness on youth, the present study examined a moderated mediation model of the effects of parental illness on youth and family functioning derived from the Family Ecology Framework (FEF) [21].

**Figure 1:**
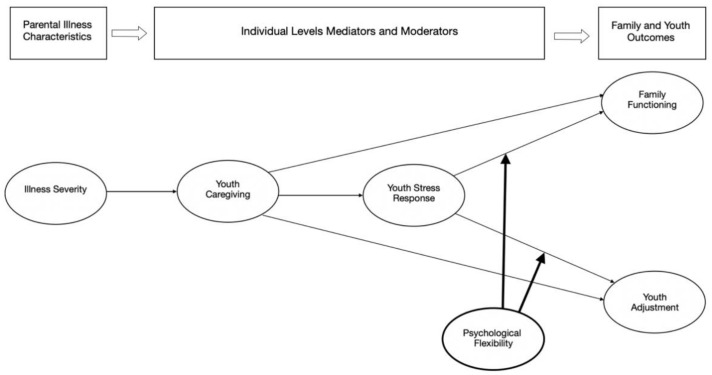
A moderated mediation model of the effects of parental illness on youth adjustment and family functioning derived from the Family Ecology Framework (FEF): the buffering effects of psychological flexibility on youth caregiving and stress.

The FEF relies on general systems, human ecology, and stress/coping theories and posits a set of mediating and buffering mechanisms linking parental illness to youth adjustment and family functioning. The FEF proposes that parental illness affects youth and family functioning indirectly through individual-level (e.g., daily hassles, youth stress, and stigma) and family-level (e.g., role redistribution) mediators. Additionally, these mediational pathways can be affected by buffering mechanisms (e.g., coping style and psychological resources). Pedersen and Revenson [21] reviewed the evidence supporting links between individual FEF elements, and model testing research provided preliminary support for the overall framework (e.g., [10]). For the purposes of the present study, we derived a model from the FEF with the following components: parental illness severity, two mediators (youth caregiving and stress), a buffering mechanism (youth psychological flexibility), and the outcomes, youth adjustment, and family functioning. The model is summarized in Figure 1, and each of the components is discussed below.

### 1.1. Illness Severity and Youth Adjustment and Family Functioning

According to the FEF, parental illness severity has indirect effects on youth adjustment and family functioning [21]. Irrespective of type of diagnosis, more severe parental illness has greater impacts on the parents’ ability to fulfill familial roles and responsibilities, which places higher demands on family members, and increases the risks of poorer youth adjustment and family functioning [3,5,7,13,22]. Compared to objective illness severity indicators (e.g., stage or prognosis), research indicates that higher perceived illness severity is associated with increased distress in family members [23,24]. Another indicator of parental illness severity, illness unpredictability, is associated with more worry about parental illness and increased anxiety and depressive symptoms in family members [22,25,26].

### 1.2. Mediators of the Effects of Parental Illness on Youth Adjustment and Family Functioning

The FEF proposes that parental illness affects youth and family functioning indirectly through various youth responses to parental illness. For this study, we focused on two of these mediating processes: role redistribution (operationalized at the individual level as youth caregiving) and youth stress. According to the FEF [21], because of the redistribution of family roles related to parental illness, youth tend to assume more caregiving responsibilities and take on more caregiving tasks, including basic domestic duties (e.g., laundry, cooking, cleaning), practical activities of daily living (e.g., managing finances and supervising medications), personal care tasks (e.g., toileting, changing dressings, and assisting with mobility), and providing emotional support to the ill parent (e.g., ensuring the ill parent is happy, gainfully occupied, and safe) [13,17,27,28]. According to the FEF, parental illness also leads to increases in perceived stress. Two studies found support for youth caregiving and stress as mediators of the effects of parental illness on youth and family functioning [10] and youth internalizing problems [22]. Research also showed that parental illness was associated with higher youth caregiving [9,10,11,12,13,14,17,29] and stress [7,10,14,18,20,30]. In the present study, we examined youth caregiving and stress as serial mediators and tested the moderating effects of youth psychological flexibility on these mediating mechanisms. 

### 1.3. Psychological Flexibility as Moderator of the Effects of Parental Illness on Youth Adjustment and Family Functioning

The FEF includes numerous potential moderating mechanisms, the presence of which can buffer or intensify the detrimental impacts of parental illness. Buffering mechanisms are hypothesized to ameliorate the negative effects of the youth stress response on youth and family functioning. One such mechanism is psychological flexibility, defined as the ability to effectively manage unhelpful thoughts and emotional discomfort in the present without expending effort to change them, while at the same time engaging in behavior to pursue personal values, thereby enabling optimal adaptation to changing circumstances [31]. For example, psychological flexibility in youth growing up with a parent affected by chronic illness may involve noticing with acceptance unhelpful thoughts in the present that are associated with their parent’s illness and related stressors, without investing energy in changing them, and instead diverting attention to engagement in valued activities (e.g., playing a cherished sport), leading to greater fulfillment. Psychological flexibility is a cornerstone of psychological health [32] and is the overarching construct that underpins the most widely researched third wave cognitive behavior therapy (CBT) called acceptance and commitment therapy (ACT) [31]. We found no published studies on the stress-buffering effects of psychological flexibility in youth and families experiencing parental chronic illness. However, in a sample of youth, psychological flexibility moderated the relationships between daily stress and physical and mental health outcomes [33]. At the familial level, psychological flexibility has been found to moderate the relationship between parent and child distress [34]. Psychological flexibility has also been shown to moderate the adverse effects of major stressful life events on mental health [35]. Testing the proposed buffering role of psychological flexibility within the FEF has the potential to yield results that can inform the development of ACT-based interventions that promote youth and family functioning in the context of parental illness.

### 1.4. The Present Study

The aim of the present study was to test a moderated mediation model derived from the FEF, which proposed that youth caregiving and stress were serial mediators of the impacts of parental illness on youth adjustment and family functioning and that psychological flexibility moderated the effects of these mediational mechanisms. Consistent with this model, we hypothesized that (1) youth caregiving and stress would serially mediate the adverse impacts of parental illness on youth adjustment and family functioning, and (2) psychological flexibility would buffer the negative effects of the final mediation link between youth stress and the outcomes (youth adjustment and family functioning).

## 2. Materials and Methods

### 2.1. Participants and Recruitment Procedure

A total of 387 youth of parents affected by chronic illness participated in this cross-sectional study. Participants were recruited across Italy via brochures and posters in schools, illness-related local community organizations (e.g., cancer, epilepsy, and multiple sclerosis), and waiting rooms of health facilities as well as via posting on social networks. The study was advertised as “The Promotion of Mental Health and Wellbeing in Youth Project” and targeted youth living with an ill parent. Eligibility criteria included living with a parent affected by a serious medical condition or disability and aged 11 to 24 years. Exclusion criteria were an insufficient command of Italian, cognitive impairments, and severe medical conditions in youth themselves or other non-parent family members. The study was approved by the University of Bologna ethics committee. A researcher administered the hard copy questionnaire face-to-face, usually at the family home, after obtaining active informed consent from both parents if youth were underage, or from youth themselves if they were ≥18 years old. The variation in recruitment methods precluded the calculation of an overall response rate.

Overall, the average percentage of missing data was low (0.25%). Following the guidelines of Darlington and Hayes [36], five cases were identified as outliers using t-residual distributions. The exclusion of these outliers did not change the results of the primary analyses; hence, analyses were reported using the full sample.

### 2.2. Measures

All measures used in the present study have been validated for use with adolescents and young adults.

#### 2.2.1. Demographic Characteristics

Youth indicated their age (via the date of birth), gender, education, employment (Do you have a paid part-time job?), nationality, number of family members, number of siblings, and dual- or single-parent family status. Youth also indicated which parent had a health condition (mother, father, both) and named the chronic illness of the ill parent.

#### 2.2.2. Parental Illness Severity

Parental illness severity was assessed by scales developed, validated, and used in prior published research in this field (e.g., [9,10,11,12,13,14,25,37]). Perceived illness severity: youth rated the seriousness of their parent’s health condition on a 5-point scale (1 not at all serious to 5 very serious). Illness unpredictability: youth reported the extent to which they agreed with 5 items examining parental illness unpredictability (e.g., My parent’s condition could change at any time with little warning). Items were rated on a 5-point scale (1 strongly disagree to 5 strongly agree). In order to create an index of parental illness severity for this study, we averaged scores of perceived illness severity and illness unpredictability, with higher index scores reflecting greater illness severity. The observed Cronbach’s alpha for the index of parental illness severity was excellent (α = 0.91).

#### 2.2.3. Youth Caregiving

Youth caregiving was assessed by the Caregiving Responsibilities subscale of the Italian version [38] of the Young Carer of Parents Inventory-Revised [25,37]. It consisted of 7 items (e.g., My parent(s) relies on me to help them with household chores and My parent(s) expects me to help care for them) rated on a 5-point scale (0 strongly disagree to 4 strongly agree). Scores were averaged with higher scores reflecting greater caregiving responsibilities. The subscale demonstrated good internal reliability and validity in the derivation [25,37] and Italian validation studies [38]. The caregiving responsibilities subscale was used as an independent predictor in prior young carers research [9,10,11,12,13,39,40]. The observed Cronbach’s alpha was good (α = 0.78).

#### 2.2.4. Youth Stress

The Chronic Stress Questionnaire for Children and Adolescents (CSQ-CA) [41] was used to assess youth stress. It consisted of 17 items measuring chronic stress in the past three months (e.g., I easily overreact to situations and I often feel relaxed). Items were rated on a 4-point scale (1 not true for me at all to 4 completely true for me). Scores were summed with higher scores indicating higher stress. The CSQ-CA demonstrated good reliability and convergent and divergent validity [41]. Because the CSQ-CA has not been validated in Italian, we ran a Confirmatory Factor Analysis (CFA). Fit indices of the CFA of the Italian CSQ-CA were satisfactory for the original one-factor model: χ^2^ (119) = 151.620, *p* < 0.001; CFI = 0.959; TLI = 0.930; RMSEA = 0.047; RMSEA 90%; CI = 0.036, 0.059. The observed Cronbach’s alpha was good (α = 0.82).

#### 2.2.5. Psychological Flexibility

The Italian version [42] of the short-form Avoidance and Fusion Questionnaire for Youth (AFQ-Y8) [43] was used to assess psychological flexibility. It consisted of 8 items (e.g., My life won’t be good until I feel happy and My thoughts and feelings mess up my life) rated on a 5-point scale (0 not at all true to 4 very true). Items are scored in the direction of psychological inflexibility. For this study, a total score was obtained by summing the reverse ratings on all items, such that higher scores indicated higher psychological flexibility. The scale demonstrated good reliability and validity [44]. The observed Cronbach’s alpha was good (α = 0.79).

#### 2.2.6. Youth Adjustment—Internalizing and Externalizing Problems

To assess negative youth adjustment outcomes, the internalizing and externalizing problem scales of the Italian version [45] of the Youth Self-Report (YSR) [46] were used. The YSR internalizing scale consisted of three factors: anxious/depressed, withdrawn/depressed, and somatic. The YSR externalizing scale reflected two factors: rule-breaking behaviors and aggressive behaviors. Items were rated on a 3-point scale (0 not true to 2 very true). Scores were summed with higher scores reflecting more problems. The original YSR demonstrated sound psychometric proprieties, including test-retest reliability, internal consistency, and content, criterion-related, and construct validity [46]. It was also used with youth aged 10–25 years [20,47] and evinced metric age measurement invariance in a previous study [39]. The observed Cronbach’s alphas for internalizing and externalizing problems were good (α_s_ = 0.90 and 0.84, respectively).

#### 2.2.7. Youth Adjustment—Psychological Wellbeing

To assess positive youth adjustment outcomes, the psychological wellbeing scale of the Kidscreen-27 [48,49] was employed. The Kidscreen-27 assessed youth HRQoL across five dimensions (i.e., physical wellbeing, psychological wellbeing, autonomy/parent relations, peers/social support, and school environment). The psychological wellbeing subscale consisted of 7 items (e.g., Have you felt fit and well? and Have you been able to rely on your friends?) rated on a 5-point Likert scale (0 not at all to 4 extremely or 0 never to 4 always). Total scores were calculated by summing all items, with higher scores indicating greater psychological wellbeing. The Kidscreen-27 was validated in a large population-based sample of youth from several European countries, including Italy, as well as in a sample of youth whose parents had a range of physical and mental conditions [6,49]. It was used with young people aged 16–35 years [50], and it demonstrated metric age measurement invariance in a previous study [39]. The observed Cronbach’s alpha was good (α = 0.88).

#### 2.2.8. Family Functioning

The Italian 12-item general family functioning subscale [51] of the Family Assessment Device (FAD) [52] was used to assess family functioning (e.g., Planning family activities is difficult because we misunderstand each other and We don’t get along well together). Items were rated on a 5-point Likert scale (0 strongly agree to 4 strongly disagree). Scores were averaged, with higher scores indicating poorer family functioning. The FAD is a widely used measure of family functioning and has demonstrated good reliability and validity [53]. The observed Cronbach’s alpha was good (α = 0.90).

### 2.3. Data Analysis Approach

Preliminary analyses (i.e., descriptive statistics, reliabilities, and correlations among study variables) were conducted in IBM SPSS 24. Demographics significantly correlated with youth adjustment and family functioning were controlled for in the path analyses. Path analyses were conducted using the Process macro v.3.5 [54] with Model 6 (two serial mediators) and Model 87 (two serial mediators and a second stage moderator). For each model, we conducted four analyses, one for each dependent variable (i.e., Y_1_ = internalizing problems, Y_2_ = externalizing problems, Y_3_ = psychological wellbeing, and Y_4_ = family functioning). As depicted in Figure 1, we tested a model in which illness severity (X = independent variable) would indirectly influence youth adjustment and family functioning (Y_1_–Y_4_) through causally linked serial mediators: youth caregiving (M_1_ = mediator 1) and youth stress (M_2_ = mediator 2). Within these serial mediating processes, we examined a moderated mediation or conditional process model combining the estimation of the conditional nature (the second stage moderation effect of psychological flexibility; W = moderator) of the serial indirect effects of illness severity on youth adjustment and family functioning. In other words, we tested whether the process through which illness severity affects youth adjustment and family functioning via youth caregiving and youth stress, was conditional on psychological flexibility (W). Mediation was established by computing 95% bootstrap confidence intervals (CIs) with 5000 random bootstrap samples for each indirect effect. Statistical significance of each indirect effect was established when zero was not included within the lower and upper levels of the 95% CIs. Moderation was assessed by testing the presence of a significant interaction between youth stress and psychological flexibility (M_2_ × W) on each dependent variable. In order to test the moderation of the serial indirect effect—i.e., to examine whether the serial indirect effect via youth caregiving (M_1_) and youth stress (M_2_) was conditional on psychological flexibility (W)—we calculated the index of moderated mediation [55]. This index represented a direct quantification of the linear association between the moderator (W) and the indirect effect. In our analyses, the index of moderated mediation quantified whether psychological flexibility moderated the serial indirect effect through youth caregiving (M_1_) and youth stress (M_2_). It represented the slope of the linear relationship between psychological flexibility (W) and the serial indirect effect and quantified how much the serial indirect effect changed as psychological flexibility changed. The moderated mediation was established when the 95% bootstrap CI of the index did not include zero. In each model, we calculated the unstandardized coefficients representing the amount by which the dependent variable or mediator would change if we changed a given predictor by one unit controlling for all the other variables included in the model. Further information on how to interpret the unstandardized coefficients, the serial indirect effect, and the index of moderated mediation is reported in the Supplementary Materials.

## 3. Results

### 3.1. Participant Characteristics

Participant characteristics and descriptive data for the study variables are reported in Table 1. The mean age of participants was 17.71 years (*SD* = 3.61), with 40.8% being male. Most (83.9%) were currently studying, while 29% had part-time jobs. Almost all youth (99%) were native Italian. Participants indicated an average family size of 4.04 members (*SD* = 1.14), with a mean number of siblings of 1.27 (*SD* = 0.39). Most participants lived in a dual-parent family, with 6.2% living in a single-parent family. Of the ill parents, 63.3% were female (30% male), while 6.7% of youth reported both parents had a serious medical condition or disability. Parental illnesses were classified according to the International Classification of Diseases 11th Revision (ICD-11) into cancer (34.6%), neurological diseases (25.3%), Type I and II diabetes (15.4%), cardiovascular diseases (4.9%), gastrointestinal diseases (3.8%), mental illnesses (3.3%), respiratory diseases (2.7%), infectious diseases (2.2%), physical disabilities and musculoskeletal diseases (2.1%), autoimmune diseases (1.6%), liver diseases (1.6%), rheumatic diseases (1.4%), and others (1.1%).

Correlations among all study variables are presented in Table 2. Of the demographics, only gender and age were significantly correlated with youth adjustment and family functioning. In particular, being male was associated with higher psychological wellbeing (*r* = 0.21 **), lower internalizing problems, and poorer family functioning (*r* = −0.27 ** and −0.11 *, respectively), while being older was related to lower psychological wellbeing (*r* = −0.16 **) and poorer family functioning (*r* = 0.13 *). Therefore, we controlled for both gender and age in the path analyses.

### 3.2. Path Analyses

**Figure 2:**
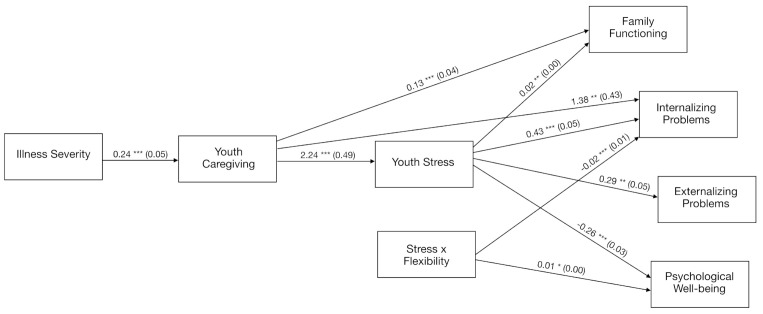
Unstandardized regression coefficients (standard errors) of the moderated mediation or conditional process path analyses depicting the buffering effects of psychological flexibility on youth caregiving and stress. Note. Youth stress and psychological flexibility were mean centered. * *p* < 0.05, ** *p* < 0.01, *** *p* < 0.001.

We conducted path analyses to test the hypothesis that youth caregiving and youth stress serially mediated the impacts of parental illness severity on youth adjustment and family functioning. We also examined the moderating effects of psychological flexibility on these serial mediating processes by testing a moderated mediation model analyzing whether the process through which illness severity affected youth adjustment and family functioning via youth caregiving and youth stress was conditional on psychological flexibility. Unstandardized coefficients with CIs for all significant paths of the final moderated mediation or conditional process models are displayed in Figure 2.

#### 3.2.1. Serial Mediation of Youth Caregiving and Stress on the Link between Parental Illness and Youth Adjustment and Family Functioning

Results indicated that parental illness severity did not directly predict internalizing problems (coeff. = 0.212, *SE* = 0.446, 95% bootstrap CI = −0.664, 1.089), externalizing problems (coeff. = 0.507, *SE* = 0.400, 95% bootstrap CI = −0.279, 1.292), psychological wellbeing (coeff. = −0.280, *SE* = 0.273, 95% bootstrap CI = −0.816, 0.256), or family functioning (coeff. = 0.040, *SE* = 0.028, 95% bootstrap CI = −0.016, 0.096). Rather, parental illness severity exhibited a significant indirect effect via youth caregiving, predicting higher internalizing problems (coeff. = 0.448, *SE* = 0.129, 95% bootstrap CI = 0.122, 0.622) and poorer family functioning (coeff. = 0.014, *SE* = 0.006, 95% bootstrap CI = 0.001, 0.024), but not externalizing problems (coeff. = 0.126, *SE* = 0.093, 95% bootstrap CI = −0.093, 0.273) and psychological wellbeing (coeff. = 0.019, *SE* = 0.057, 95% bootstrap CI = −0.051, 0.174). Furthermore, parental illness severity did not display a significant indirect effect via youth stress on internalizing problems (coeff. = 0.028, *SE* = 0.409, 95% bootstrap CI = −0.779, 0.850), externalizing problems (coeff. = 0.016, *SE* = 0.229, 95% bootstrap CI = −0.432, 0.480), psychological wellbeing (coeff. = −0.014, *SE* = 0.206, 95% bootstrap CI = −0.424, 0.388), or family functioning (coeff. = 0.001, *SE* = 0.016, 95% bootstrap CI = −0.026, 0.027). However, as hypothesized, parental illness severity displayed a significant indirect effect on youth adjustment and family functioning serially through youth caregiving and stress. Specifically, parental illness severity predicted higher internalizing problems (coeff. = 0.399, *SE* = 0.121, 95% bootstrap CI = 0.191, 0.660) and externalizing problems (coeff. = 0.223, *SE* = 0.069, 95% bootstrap CI = 0.105, 0.373), poorer family functioning (coeff. = 0.013, *SE* = 0.004, 95% bootstrap CI = 0.006, 0.224), and lower psychological wellbeing (coeff. = −0.200, *SE* = 0.062, 95% bootstrap CI = −0.333, −0.093) serially via youth caregiving and stress. Unstandardized coefficients with CIs for each serial mediation model estimating youth adjustment and family functioning are reported in Table S2 in the Supplementary Materials.

#### 3.2.2. Moderated Mediation: The Buffering Effects of Psychological Flexibility through Youth Caregiving and Stress

Unstandardized coefficients with CIs for each moderated mediation model estimating youth adjustment and family functioning are reported in Table 3 and displayed in Figure 2. Results of the path analyses revealed that psychological flexibility moderated the final mediation link between stress and both internalizing problems (stress * psychological flexibility = −0.023, *SE* = 0.007, *p* < 0.001) and psychological wellbeing (stress * psychological flexibility = 0.011, *SE* = 0.004, *p* < 0.05) but not externalizing problems (0.006, *SE* = 0.006, *p* > 0.05) and family functioning (0.000, SE = 0.001, *p* > 0.05). Most importantly, results of the moderated mediation analyses showed that the serial indirect effect of parental illness severity on youth internalizing problems and psychological wellbeing, through youth caregiving and stress, varied as a function of psychological flexibility with internalizing problems (index of moderated mediation of the serial indirect effect through M_1_ and M_2_ = −0.013, *SE* = 0.006, 95% CI −0.025, −0.004) and psychological wellbeing (index of moderated mediation of the serial indirect effect through M_1_ and M_2_ = 0.006, *SE* = 0.005, 95% bootstrap CI = 0.000, 0.008) but not with externalizing problems (0.003, *SE* = 0.004, 95% bootstrap CI = −0.005, 0.012) and family functioning (−0.000, *SE* = 0.000, 95% bootstrap CI = −0.001, 0.001). Moderated mediation was therefore established only for internalizing problems and psychological wellbeing. In other word, the serial indirect effect of illness severity on internalizing problems and psychological wellbeing through youth caregiving and stress was conditional on psychological flexibility only for internalizing problems and psychological wellbeing.

**Figure 3:**
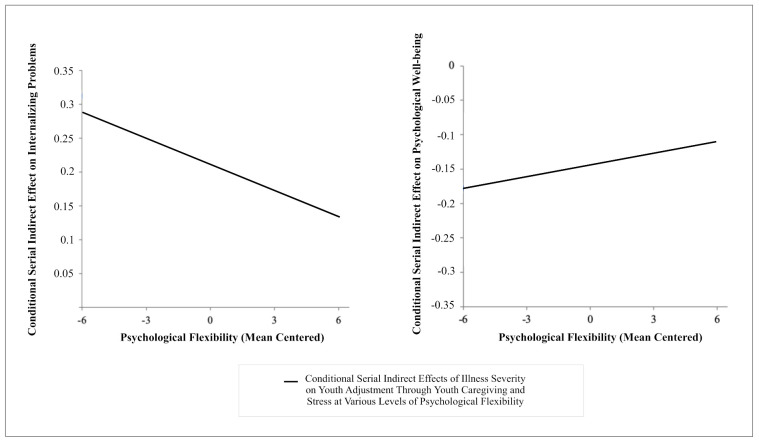
A visual representation of the linear functions relating psychological flexibility to the serial indirect effects of illness severity on internalizing problems and psychological wellbeing via youth caregiving and stress. Note. Results are depicted controlling for the control variables (age and gender) included in the moderated mediation models.

A visual representation of how much the serial indirect effect of illness severity on internalizing problems and psychological wellbeing through youth caregiving and stress changes as a function of changes in psychological flexibility is depicted on Figure 3. As displayed, for internalizing problems with higher psychological flexibility the serial indirect effect of parental illness via youth caregiving and stress becomes lower and is associated with lower internalizing problems, while for psychological wellbeing with higher psychological flexibility the serial indirect effect of parental illness via youth caregiving and stress becomes higher and is associated with higher psychological wellbeing. In summary, with respect to the second prediction, results supported the proposed buffering role of youth psychological flexibility on youth caregiving and stress, by way of mitigating the adverse effects of parental illness on youth internalizing problems and psychological wellbeing. 

## 4. Discussion

The present study examined a moderated mediation model of the effects of parental illness on youth adjustment and family functioning derived from the FEF [21]. Consistent with this model, we tested two predictions: (1) that youth caregiving and stress were serial mediators of the adverse impacts of parental illness on youth adjustment and family functioning, and (2) that psychological flexibility moderated the serial indirect effects of these mediational mechanisms. The results of the path analyses supported both predictions.

Regarding the first prediction, results showed that youth caregiving and stress serially mediated the adverse effects of parental illness severity on youth adjustment and family functioning. These results are consistent with and expanded on prior findings, which supported youth caregiving and stress as individual mediators of the impacts of parental illness on youth and family outcomes [10,22]. Results of the present study suggest that parental illness severity is associated with the redistribution of family roles, whereby youth engage in more youth caregiving, which is related to higher stress in youth and, in turn, poorer youth and family outcomes. Prior research shows that when a parent has an illness, the extra caregiving responsibilities that youth assume is associated with isolation and low social support [25,29,39]. Youth caregiving also competes with other activities (e.g., school, work, and leisure), taxing youth resources and coping mechanisms [10,30]. Hence, youth caregiving and the associated demands are appraised as stressful by youth, and in turn, higher stress adversely affects youth and family outcomes [14].

With respect to the second prediction, results supported the proposed buffering role of youth psychological flexibility on youth caregiving and stress by way of mitigating the adverse effects of parental illness on youth internalizing problems and psychological wellbeing. These findings are notable given this is the first published study to examine the protective role of psychological flexibility for youth in the context of parental illness. The stress-buffering effects of psychological flexibility were evident for both positive (psychological wellbeing) and negative (internalizing problems) youth adjustment outcomes. These results are consistent with those from prior research showing that psychological flexibility buffers the detrimental effects of adult caregiver distress [56].

Psychological flexibility did not have a stress-buffering effect on youth externalizing problems and family functioning. The absence of significant buffering effects on family functioning might be due to the assessment of psychological flexibility at the individual level in youth. A family-level assessment of psychological flexibility (e.g., in multiple family members) might evidence stronger stress-buffering effects of psychological flexibility on family functioning.

With respect to externalizing problems, the total variance accounted for by the model predicting externalizing problems was lower (29%) than that for internalizing problems (61%). Consistent with previous studies, parental illness seemed to have a stronger impact on youth internalizing problems than externalizing problems (e.g., see review, [15]), which potentially reduced the scope for psychological flexibility to play a stress-buffering role. Mirroring the pattern of results in the present study, other studies found a stronger relationship between psychological flexibility and internalizing symptoms and a weaker relationship between psychological flexibility and externalizing symptoms in youth [44,57]. Hence, it is also possible that psychological flexibility differentially affected youth with predominantly internalizing (e.g., depression and anxiety) vs. externalizing problems (e.g., aggression and oppositional/conduct problems). However, given this area of inquiry is relatively new and the psychological flexibility findings of the present study are therefore preliminary, it is not possible to draw firm conclusions on why psychological flexibility differentially impacted internalizing and externalizing problems in youth.

In view of the detrimental mental health, social, developmental, and educational impacts of parental illness on youth adjustment and the disruptive effects on family functioning [3,5,7,9,10,11,12,13,14,15,19,20], it is essential that effective public health interventions are developed to bolster resilience and promote wellbeing in affected youth and families. Our findings identified three potential intervention targets: youth caregiving (and associated caregiving responsibilities), related stress appraisals, and psychological flexibility.

Psychological flexibility, in particular, provides an empirically supported intervention pathway via ACT which shows potential in addressing the psychosocial and mental health vulnerabilities of youth and families in the context of parental illness. ACT is an empirically supported intervention approach that fosters psychological flexibility [31,58,59]. The ACT psychological flexibility framework specifies six therapeutic processes that promote psychological flexibility: (1) acceptance—openness to experience, (2) cognitive defusion—observing thoughts rather than taking them literally, (3) present moment awareness (mindfulness)—open and responsive awareness of the present, (4) self-as-context—flexible self-awareness and perspective-taking, (5) values—freely chosen personally meaningful life directions, (6) committed action—values-guided effective action.

Results from the present study suggested that the six ACT processes used to foster psychological flexibility could be tailored to help youth effectively balance their caregiving responsibilities in relation to other valued pursuits and manage their perceptions of care-giving stress [60]. Evidence shows that ACT is effective in improving psychological flexibility and mental health outcomes in clinical and non-clinical youth and adult populations (see the review of meta-analyses, [58]). In other research, psychological flexibility was also associated with decreases in stress in adult carers [60,61]. A meta-analysis of ACT interventions for adult family carers found that ACT had moderate effects on depression and quality of life, small effects on anxiety, and small to moderate effects on stress [61]. Public health ACT-based interventions have also been shown to promote mental health in a variety of youth populations [62,63,64,65,66,67,68] using flexible modes of delivery: youth in the general population via group delivery [59,63,64,66], and university students via online delivery [65,67,68]. An advantage of psychological flexibility informed interventions is that they have been shown to cultivate skills that foster resilience in the context of health-related adversities, such as caregiving [61] and chronic disease (e.g., multiple sclerosis [69], diabetes [70], cancer [71]), and psychological flexibility has been shown to mediate the beneficial effects of these programs [67]. At a broader level, a whole family approach should guide the design of such interventions, given that parental illness impacts youth adjustment and family functioning in the context of complex reciprocal intimate family relations [29]. For example, such an approach might involve the provision of age and role-appropriate self-help resources to family members that guide them in the development of the six psychological flexibility processes. This would offer family members a common language and set of skills around coping with the challenges of parental illness.

This study had several methodological limitations. First, the non-random sampling increases the risk of volunteer response bias and limits the generalizability of findings. However, participants were recruited from a wide range of facilities using diverse recruitment strategies. Second, because of the cross-sectional design, it was not possible to make inferences about causal directionality among the tested components of the FEF. Third, the broad age range (11–24 years) of youth in this sample encompassed numerous developmental phases. Nevertheless, the caregiving measures used in the present study were developed and validated using samples with a similar age range. In addition, we demonstrated the age invariance of the instruments used to assess youth adjustment, and we controlled for age in the path analyses. Fourth, we did not account for parental illness duration, and there is evidence suggesting that longer parental illness duration is associated with poorer youth adjustment [15,25].

Future studies should employ longitudinal research designs to examine causal links among the FEF components over time. Further studies should also assess family-level mediators and moderators within the FEF and explore the mediating and moderating pathways within the FEF at the dyadic, family, and societal levels. Future research should explore potential interrelations among different youth developmental phases and youth adjustment within the FEF. In addition, a global measure of psychological flexibility was used in this study, and future studies should investigate the potential differential effects of the six psychological flexibility sub-processes on the outcomes. Finally, this study only assessed one youth caregiving dimension. Future research should employ the three empirically supported dimensions of youth caregiving (caregiving responsibilities, experiences, and tasks; [13,39]) and explore their role within the FEF.

## 5. Conclusions

Findings from this theory-informed study supported the predictions that youth caregiving and stress were serial mediators of the adverse impacts of parental illness on youth adjustment and family functioning and that psychological flexibility moderated the effects of these mediational mechanisms. The translational significance of this study was highlighted by its focus on families impacted by parental chronic illness and the empirical validation of mechanisms that ameliorated and exacerbated the detrimental effects of parental illness on youth and family functioning. These findings point to intervention pathways for addressing a pressing public health issue, the psychosocial and mental health vulnerabilities of youth in the context of parental illness. The evidence emerging from the literature on psychological flexibility (see the review of meta-analyses, [58]) provides strong support for the use of ACT-based interventions to promote psychological flexibility and mental health in youth who have a parent with a chronic illness.

## Figures and Tables

**Table 1:** Sample characteristics and descriptive data for the study variables (N = 387).

	%	M (*SD*)	Range
*Sample characteristics*			
Age in years		17.71 (3.61)	11–24
Gender: male	40.8		
Currently studying	83.9		
Current part-time job	29.0		
Family size		4.04 (1.14)	2–8
Single-parent family	6.2		
*Descriptive data for study variables*			
Illness Severity		2.62 (0.83)	1–5
Youth Caregiving		1.50 (0.78)	0–3.86
Stress		42.45 (8.01)	21–60
Psychological Flexibility		22.88 (5.71)	5–32
Internalizing Problems		15.52 (9.76)	0–42
Externalizing Problems		10.04 (6.48)	0–34
Psychological Wellbeing		26.01 (5.02)	8–35
Family Functioning		1.93 (0.55)	1–4

**Table 2:** Correlations among study variables (N = 387).

	1	2	3	4	5	6	7	8
1. Illness Severity	−							
2. Youth Caregiving	0.24 **	−						
3. Stress	0.10 *	0.24 **	−					
4. Psychological Flexibility	−0.07	−0.22 **	−0.51 **	−				
5. Internalizing Problems	0.12 *	0.29 **	0.56 **	−0.59 **	−			
6. Externalizing Problems	0.10	0.18 **	0.45 **	−0.43 **	0.55 **	−		
7. Psychological Wellbeing	−0.11 *	−0.14 **	−0.52 **	0.52 **	−0.66 **	−0.35 **	−	
8. Family Functioning	0.23 **	0.20 **	0.37 **	−0.32 **	0.49 **	0.39 **	−0.49 **	−
9. Gender (0 = female)	−0.08	−0.02	−0.32 **	0.14 **	−0.27 **	0.04	0.21 **	−0.11 *
10. Age	0.16 **	0.10	0.16 **	0.03	0.01	0.01	−0.16 **	0.13 *

Note. * *p* < 0.05, ** *p* < 0.01. Pearson’s correlation for continuous variables and point-biserial correlations for categorical variables.

**Table 3:** Unstandardized coefficients with confidence intervals of each moderated mediation model estimating youth adjustment and family functioning.

	Youth Caregiving (*M*_1_)		Youth Stress (*M*_2_)		Internalizing Problems (*Y*_1_)		Externalizing Problems (*Y*_2_)		Psychological Wellbeing (*Y*_3_)		Family Functioning (*Y*_4_)	
	Coeff. (SE)	95% CI	Coeff. (SE)	95% CI	Coeff. (SE)	95% CI	Coeff. (SE)	95% CI	Coeff. (SE)	95% CI	Coeff. (SE)	95% CI
Illness Severity (*X*)	0.244 ***,	0.139,	0.038	−1.007,	0.212	−0.664,	0.507,	−0.279,	−0.280	−0.816,	0.047	−0.021,
	(0.054)	0.350	(0.531)	1.083	(0.446)	1.089	(0.400)	1.292	(0.273)	0.256	(0.035)	0.115
Youth Caregiving (*M*_1_)			2.242 ***	1.272,	1.375 ***	0.535,	0.325,	−0.428,	0.236,	−0.278,	126 ***,	0.054,
			(0.494)	3.213	(0.427)	2.216	(0.383)	1.079	(0.261)	0.750	0.036	0.197
Youth Stress (*M*_2_)					0.433 ***,	0.332,	0.291 **,	0.201,	−0.263 ***,	−0.324,	0.018 **,	0.010,
					(0.051)	0.533	(0.046)	0.381	(0.031)	−0.202	(0.004)	0.026
Psychological Flexibility (*W*)					−0.738,	−0.874,	−0.293,	−0.415,	0.256,	0.172,	−0.014,	−0.025,
					(0.069)	−0.601	(0.062)	−0.171	(0.042)	0.339	(0.006)	0.003
Interaction (*M*_2_ × *W*)					−0.023 ***,	−0.037,	0.006,	−0.007,	0.011 *,	0.002,	0.000,	−0.001,
					(0.007)	−0.010	(0.006)	0.018	(0.004)	0.019	(0.001)	(0.001)
Gender (*U*_1_)	0.001,	−0.154,	−5.061 ***,	−6.557,	−1.792 **,	−3.121,	2.495 ***,	1.304,	0.242	−0.571,	0.015,	−0.093
	(0.079)	0.155	(0.761)	−3.566	(0.676)	−0.463	(0.606)	3.687	(0.414)	1.055	(0.055)	(0.123)
Age (*U*_2_)	0.013,	−0.008,	0.288 **,	0.082,	−0.201 *,	−0.376,	−0.109,	−0.266,	−0.121 *,	−0.228,	0.008,	−0.006,
	(0.011)	0.034	(0.105)	0.493	0.089	−0.027	(0.08)	0.048	(0.054)	−0.014	(0.007)	0.022
	R^2^ = 0.061 ***		R^2^ = 0.172 ***		R^2^ = 0.611 ***		R^2^ = 0.290 ***		R^2^ = 0.449 ***		R^2^ = 0.197 ***	
	*F*(3, 383) = 8.235		*F*(4, 382) = 19.806		*F*(7, 379) = 84.912		*F*(7, 379) = 22.159		*F*(7, 379) = 44.074		*F*(7, 379) = 13.296	
Index of Moderated Mediation					**Coeff. (SE)**	**95% CI**	**Coeff. (SE)**	**95% CI**	**Coeff. (SE)**	**95% CI**	**Coeff. (SE)**	**95% CI**
Moderation of the serial indirect effect through Caregiving (*M*_1_) and Stress (*M*_2_)					−0.013,	0.025,	0.003,	−0.005,	0.006,	0.000,	−0.000,	−0.001,
					(0.006)	−0.004	(0.004)	0.012	(0.005)	0.008	0.000	0.001

Note. * *p* < 0.05, ** *p* < 0.01, *** *p* < 0.001. Coeff. = unstandardized regression coefficient; SE = standard error; X = independent variable; *M*_1_, *M*_2_ = first and second mediators; *W* = moderator; *M*_2_ × *W =* interaction between second mediator and moderator; *U*_1_, *U*_2_ = control variables, *Y*_1_, *Y*_2_, *Y*_3_, *Y*_4_ = dependent variables. Youth stress and psychological flexibility were mean centered.

## Data Availability

The dataset analyzed during the current study are available from the corresponding author upon reasonable request.

## Supplementary Materials

The following are available online at https://www.mdpi.com/article/10.3390/ijerph18094902/s1, Figure S1: Interpretation of unstandardized coefficients in Process model 6 and 87. Table S2: Unstandardized coefficients with confidence intervals of each serial mediation model estimating youth adjustment and family functioning.
