# Supplementary material for: A Model of the Effects of Parental Illness on Youth Adjustment and Family Functioning: The Moderating Effects of Psychological Flexibility on Youth Caregiving and Stress
Source: Int J Environ Res Public Health. 2021 May 4;18(9):4902. doi: 10.3390/ijerph18094902 (PMC8124913; doi:10.3390/ijerph18094902)
Supplement: Supplementary file 1 [file ijerph-18-04902-s001.zip › Supplementary Materials 1.pdf]

Interpretation of Unstandardized Coefficients in Process Model 6

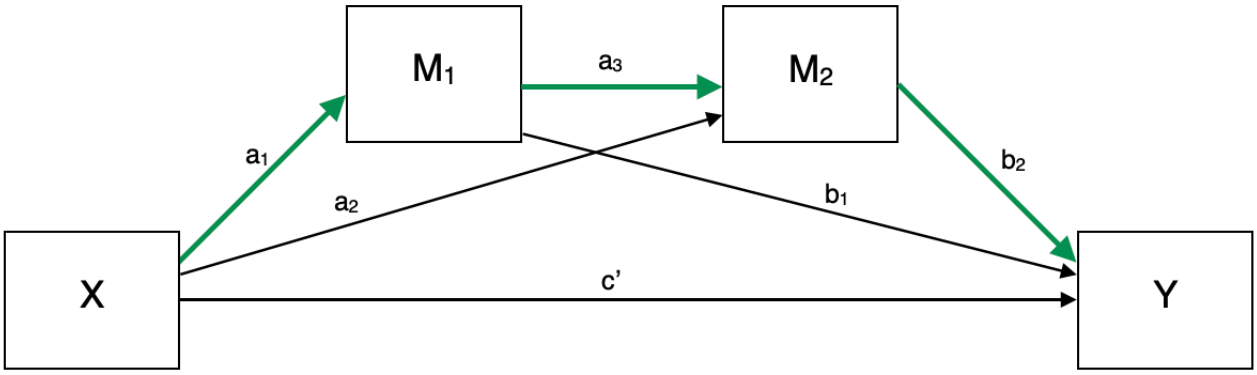

Note. X = independent variable; M_1, M_2 = first and second mediators; Y = dependent variable. For ease of interpretation the two control variables (U_1 and U_2) that are regressed on all variables included in the model are not depicted in the figure. Adapted and modified from Hayes 2018.

a_1 = the effect of X on M_1 controlling for the control variables (U_1 and U_2)

a_2 = the effect of X on M_2 controlling for the other mediator (M_1) and the control variables (U_1 and U_2)

a_3 = the effect of M_1 on M_2 controlling for X and the control variables (U_1 and U_2)

b_1 = effect of M_1 on Y controlling for the other mediator (M_2), X and the control variables (U_1 and U_2)

b_2 = effect of M_2 on Y controlling for the other mediator (M_1), X and the control variables (U_1 and U_2)

c' = direct effect of X on Y controlling for M_1, M_2 and the control variables (U_1 and U_2)

Regression Equations (for ease of interpretation the two control variables, U_1 and U_2, that are regressed on all variables included in the model are not indicated in regression equations):

$M_1 = i_{M1} + a_1X$

$M_2 = i_{M2} + a_2X + a_3M_1$

$Y = i_Y + c'X + b_1M_1 + b_2M_2$

Indirect effects:

Specific indirect effect of X through $M_1 = a_1b_1$

Specific indirect effect of X through $M_2 = a_2b_2$

Serial indirect effect of X through M_1 and $M_2 = a_1a_3b_2$

Interpretation of Unstandardized Coefficients in Process Model 87

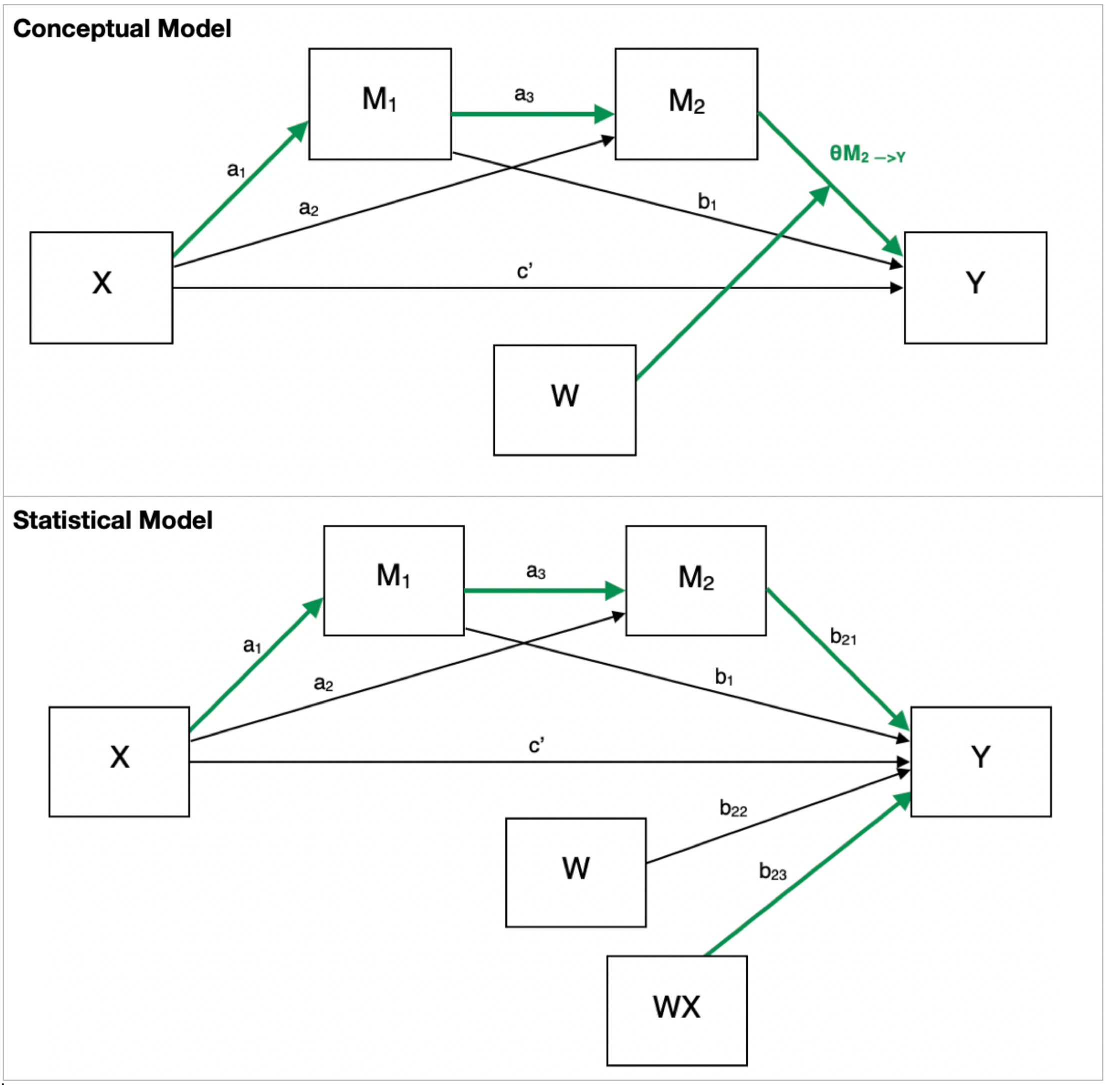

Note. X = independent variable; M_1, M_2 = first and second mediators; *W* = moderator ; U_1, U_2 = control variables, Y = dependent variable. For ease of interpretation the two control variables (U_1 and U_2) that are regressed on all variables included in the model are not depicted in the figure. Adapted and modified from Hayes 2015, 2018.

$\theta M_{2 \to Y}$ = the conditional effect of M_2 on Y (i.e., the effect of M_2 on Y is a function of W) = $(b_{21} + b_{23}W)$

Regression Equations (for ease of interpretation the two control variables, U_1 and U_2, that are regressed on all variables included in the model are not indicated in regression equations):

$M_1 = i_{M1} + a_1X$

$M_2 = i_{M2} + a_2X + a_3M_1$

$Y = i_Y + c'X + a_1M_1 + \theta M_{2 \to Y}$ or $Y = i_Y + c'X + a_1M_1 + b_{21}M_2 + b_{22}W + b_{23}M_2W$

$\rightarrow Y = i_Y + c'X + a_1M_1 + (b_{21} + b_{23}W)M_2 + b_{22}W \rightarrow \theta M_{2 \to Y} = (b_{21} + b_{23}W)$

ωM_1M_2 = Conditional serial indirect effect (i.e. the serial indirect effect of X on Y through M_1 and M_2 is a function of W) = $a_1 a_3 \theta M_{2 \to Y} \rightarrow a_1a_3(b_{21} + b_{23}W) \rightarrow a_1a_3b_{21} + \underline{a_1a_3b_{23}}W$

Index of Moderated Mediation of the serial indirect effect (through M_1 and M_2)= $\underline{a_1a_3b_{23}}$

References

Hayes, A.F. Introduction to Mediation, Moderation, and Conditional Process Analysis: A Regression-based Approach. 2nd ed. New York, NY: Guilford Press, **2018**.

Hayes, A.F. An index and test of linear moderated mediation. *Multivariate Behav Res*. **2015**, *50*, 1–22. https://doi.org/10.1080/00273171.2014.962683
