# Supplementary material for: A Model of the Effects of Parental Illness on Youth Adjustment and Family Functioning: The Moderating Effects of Psychological Flexibility on Youth Caregiving and Stress
Source: Int J Environ Res Public Health. 2021 May 4;18(9):4902. doi: 10.3390/ijerph18094902 (PMC8124913; doi:10.3390/ijerph18094902)
Supplement: Supplementary file 1 [file ijerph-18-04902-s001.zip › Supplementary Materials 2.pdf]

Supplementary Materials 2. Unstandardized coefficients with confidence intervals of each serial mediation model estimating youth adjustment and family functioning.

	**Youth Caregiving (M_1)**		**Youth Stress (M_2)**		**Internalizing Problems (Y_1)**		**Externalizing Problems (Y_2)**		**Psychological Well-being (Y_3)**		**Family Functioning (Y_4)**	
	**Coeff. (SE)**	**95 % CI**	**Coeff. (SE)**	**95 % CI**	**Coeff. (SE)**	**95 % CI**	**Coeff. (SE)**	**95 % CI**	**Coeff. (SE)**	**95 % CI**	**Coeff. (SE)**	**95 % CI**
Illness Severity (X)	.244*** (.054)	.139 .350	.038 (.531)	-1.007, 1.083	.258 (.528)	-.780, 1.296	.514 (.410)	-.292, 1.321	-.297 (.291)	-.869, .274	.056 (.035)	-.013, .124
Youth Caregiving (M_1)			2.242*** (.494)	1.272, 3.213	.1.838*** (.503)	.848, 2.828	.517 (.391)	-.252, 1.286	.077 (.277)	-.469, .622	.126*** .036	.054, .198
Youth Stress (M_2)					.728*** (.051)	.628, .828	.407*** (.040)	.329, .485	-.366*** (.028)	-.421, -.311	.023** (.004)	.016, .030
Gender (U_1)	.001 (.079)	-.154, .155	-5.061*** (.761)	-6.557, -3.566	-1.622* (.798)	-3.192, -.053	2.648*** (.620)	1.429, 3.867	.198 (.440)	-.668, 1.062	.020 (.055)	-.089 (.128)
Age (U_2)	.013 (.011)	-.008, .034	.288** (.105)	.082, .493	-.287** .105	-.493, -.080	-.136 (.082)	-.296, .025	-.108* (.058)	-.204, -.004	.006 (.007)	-.008, .020
	R^2 = .061*** *F*(3,383) = 8.235		R^2 = .172*** *F*(4,382) = 19.806		R^2 = .450*** *F*(5,381) = 62.458		R^2 = .249*** *F*(5,381) = 21.195		R^2 = . 369*** *F*(5,381) = 42.569		R^2 = .182*** *F*(5,381) = 13.152	

Indirect Effects	**Coeff. (SE)**	**95 % CI**	**Coeff. (SE)**	**95 % CI**	**Coeff. (SE)**	**95 % CI**	**Coeff. (SE)**	**95 % CI**
Indirect effect via M_1 only X → Youth Caregiving (*M_1*) → Y	.448 (.129)	.122, .622	.126 (.093)	-.093, .273	.019 (.057)	-.051, .174	.014 (.006)	.001, .024
Indirect effect via M_2 only X → Youth Stress (*M_2*) → Y	.028 (.409)	-.779, .850	.016 (.229)	-.432, .480	-.014 (.206)	-.424, .388	.001 (.016)	-.026, .027
Indirect effect via M_1 and M_2 in serial X → Caregiving (*M_1*) → Stress (*M_2*) → Y	.399 (.121)	.191, .660	.223 (.069)	.105, .373	-.200 (0.062)	-.333, -.093	.013 (.004)	.006, .224

Note. *$p < .05$, **$p < .01$, ***$p < .001$. X = independent variable; M_1, M_2 = first and second mediators; U_1, U_2 = control variables, Y_1, Y_2, Y_3, Y_4 = dependent variables.
